# Mind the gap: The role of mindfulness in adapting to increasing risk and climate change

**DOI:** 10.1007/s11625-017-0524-3

**Published:** 2018-01-04

**Authors:** Christine Wamsler

**Affiliations:** Lund University Centre for Sustainability Studies (LUCSUS), Lund University, Box 170, 221 00 Lund, Sweden

**Keywords:** Climate change, Inner transition, Inner transformation, Organisational mindfulness, Political mindfulness, Compassion, Sustainability, Well-being, Emotions, Urban governance, Planning, Risk reduction, Adaptation, Urban transformation, Traditional knowledge, Native knowledge, Mindful climate adaptation

## Abstract

It is becoming clear that increasingly complex global challenges cannot simply be solved by new technology or governments alone. We also need to develop new social practices and encourage a broader cultural shift towards sustainability. Against this background, this paper explores the role of mindfulness in adapting to increasing risk and climate change. Based on a literature review, it assesses current research on ‘mindful climate adaptation’, and explores how individual mindfulness is linked to climate adaptation. While in practice mindfulness-based approaches to climate adaptation have gained widespread recognition (e.g., by the United Nations), the results show that related research is scarce and fragmented. There is almost no research into the role of mindfulness in climate adaptation. At the same time, new scientific domains are opening up in cognate fields that illuminate the mindfulness–adaptation nexus from certain perspectives. These fields include: (1) disaster management; (2) individual well-being; (3) organisational management; (4) environmental behaviour; (5) social justice; and (6) knowledge production. As new concepts and approaches emerge, they require critical construct validation and empirical testing. The importance of further investigation is supported by a complementary empirical study, which shows that individual mindfulness disposition coincides with increased motivation to take (or support) climate adaptation actions. The paper concludes that mindfulness has the potential to facilitate adaptation at all scales (through cognitive, managerial, structural, ontological, and epistemological change processes) and should, therefore, become a core element in climate and associated sustainability research. Finally, it sketches the conceptual trajectories of the mindfulness–adaptation nexus and presents a pioneering, comprehensive framework for ‘mindful climate adaptation’.

## Introduction

Climate change is creating increasingly complex sustainability challenges that require new pathways and innovation (Kates et al. 2001; Sol and Wals 2015). Climate variability, climate hazards, and disasters are a manifestation of a systemic world, characterized by multiple causations, interactions, complex feedback loops, and inevitable uncertainty and unpredictability (Lang et al. 2012; IPCC 2014). However, current coordination mechanisms and problem-solving strategies appear insufficient to address these issues (Dhiman and Marques 2016; Kunze 2012; Sol and Wals 2015). At the same time, the potential influence of human beings’ ‘interiority’ for impeding or supporting climate adaptation is increasingly recognised (O’Brian and Hochachka 2011; Moser 2008; Wamsler et al. 2017). New solutions need to be found.

Inner transition is emerging as a potential new pathway (Dhiman and Marques 2016; Esbjörn-Hargens 2009; Gidley 2007; Inglis 2008; Frawley 2006; Wamsler et al. 2017). ‘Inner transition’, as used here, describes change within individuals that relate to their (expanded) consciousness and is associated with changes in values and behaviour. It is supported by indigenous, religious, or spiritual practices, such as mindfulness.

Mindfulness is intentional, non-judgmental attentiveness to the present moment. This inherent capacity of the human organism is rooted in the fundamental activities of consciousness (Baer 2003; Condon et al. 2013; Kabat-Zinn 1990), and is linked to established theories of attention, awareness, and emotional intelligence (Buss 1980; Brown et al. 2007; Carroll 2016; Goleman 2011). It is often viewed as a pre-requisite to the development of compassion, and involves a fundamental shift in the way we think about, and ultimately act on, local and global economic, social, and ecological crises (Carroll 2016; Ericson et al. 2014; Scharmer 2009/2016).

Although there is an increasing awareness of the potential to address global sustainability and climate change through inner transition, related knowledge is scarce (Dhiman and Marques 2016; Daffara 2011; Hamilton 2008; Inayatullah 2005, 2011; Koger 2015; O’Brian and; Hochachka 2011; Sandercock and Senbel 2011; Wamsler et al. 2017; Ray and Anderson 2000; Woiwode 2016). This paper addresses the gap, and explores the role of mindfulness in climate adaptation to foster sustainability at multiple levels.

Based on a literature review (described in “Methodology”), this paper assesses current research on mindful climate adaptation and how individual mindfulness may be linked to climate adaptation (see “Results”). From this, the core conceptual trajectories of the mindfulness–adaptation nexus are outlined, constituting the first comprehensive framework for—what is called here—‘mindful climate adaptation’ (see “Discussion and conclusions”).

## Methodology

The research method consisted of an in-depth qualitative literature review. The reviewed literature mainly consisted of scientific papers identified via Scopus, the Web of Science, LUBsearch, and Google Scholar. To ensure a comprehensive survey of relevant research across multiple disciplines, the search string included the following terms: (mindfulness OR mindful* OR contemplative OR compassion OR meditat*) AND (“climate change adaptation” OR (adaptation AND climate) OR “risk reduction” OR (“disaster response” OR “hazard response”) OR (“disaster recovery” OR “hazard recovery”) OR “hazard mitigation” OR names of specific hazards, such as flood OR storm OR landslide). This identified a considerable number of papers (e.g., 923 in Scopus on 11/05/2017). Irrelevant studies (false positives) were identified by screening the abstracts and removed. As the mindfulness–adaptation field is still emerging, there were a large number of false positives (e.g., only 132 out of the 923 Scopus articles were identified as potentially relevant). Other significant studies were identified using snowball sampling of the references until saturation was reached. The data analysis identified patterns in the mindfulness–adaptation nexus, related synergies, and research gaps. Literal reading and qualitative coding were used to analyse the results (Glaser and Strauss 1967; Strauss and Corbin 1998).

In parallel to this study, a quantitative survey was conducted that aimed to complement the literature review by empirically exploring how individual mindfulness is linked to climate adaptation. The survey study is presented in depth in Wamsler and Brink (2018). It took the form of a written questionnaire sent to 600 households in the coastal municipality of Lomma, in the Scania region of Sweden. This area was selected as it is expected to be one of the Swedish regions hardest hit by climate change (Hall et al. 2015; SCCV 2007). Households at risk from current and future climate change were identified based on municipal flood scenarios. The response rate was 36% (*n* = 217). Individual mindfulness disposition was assessed by four questions, adapted from the Five-Facet Mindfulness Questionnaire (FFMQ) (Baer et al. 2006). The FFMQ is a 39-item standardised instrument based on a 5-point Likert scale that measures mindfulness across five dimensions: observing, non-reacting, non-judging, acting with awareness, and describing. The selected questions had been tested and adapted to Swedish audiences (FFMQ_SWE; Lilja 2009). Attitudes to climate adaptation were assessed by self-ratings and actual actions. For the former, respondents were asked to rate, on a 5-point Likert scale, how motivating they found nine different circumstances. For the latter, a quantitative measure of the level/diversity of actual activity was created from a checklist of 14 common household adaptation actions (Wamsler and Brink 2014a, b), and a free-text option. SPSS software was used to analyse the data, and calculate correlations (Spearman’s rho) between mindfulness and the other variables. For more details, see Wamsler and Brink (2018).

## Results

While, in general, mindfulness research is rapidly growing (AMRA 2016) with a 30% annual increase (Ericson et al. 2014), this literature review shows a clear lack of scientific research on mindfulness in climate adaptation. At the same time, there are an increasing number of studies that address (often implicitly) fragments of the mindfulness–adaptation nexus. These were found at the interface between the cognate fields of: (1) disaster management; (2) individual well-being; (3) organisational management; (4) environmental behaviour; (5) social justice; and (6) knowledge production. They illuminate aspects of the mindfulness–adaptation nexus under different terms and concepts (Table 1) that relate to different phases and scales regarding the underlying cognitive, managerial, collective, structural, ontological, and epistemological processes (Tables 1, 2, 3).

**Table 1:** Research fields and focus areas that assess—directly and indirectly—the potential influence of mindfulness on climate adaptation. Related gaps are highlighted

Research field	Focus areas—researched aspects	Related gaps
Disaster management—with a focus on psychological resilience (risk perception and communication)	Psychological resilience in post-disaster contexts Post-traumatic stress reduction Post-disaster trauma and compassion fatigue/fade Post-disaster growth Recovery of meaning, sense of self and place Policy support for climate action Faith-based organisations and coping for response and recovery Do no harm principle Risk perception Risk and climate change communication	The link to climate change is marginal, which also explains the focus on (large-scale) disasters as opposed to small-scale events and climate variability (Wamsler 2014) Focus on agency-based solutions, rather than structural, systemic change (Wamsler et al. 2017) No adequate baseline: little knowledge of the psychological impacts of climate/natural hazards and the prevalence of related post-traumatic stress disorders (Udomratn 2009) Studies concerning stress and coping among natural/climate disaster workers are still scarce (Argentero and Setti 2011; Brown et al. 2002; Hytten and Hasle 1989) Sustainability and resilience research based on religious beliefs and spirituality are new to the traditional disaster literature (Chen n.d.) Longer term follow-ups with participants of mindfulness-based practices to assess whether the benefits of the intervention are maintained over time are missing (Hechanova et al. 2015) Religious/spiritual coping has received little attention in organisational responses following climate/natural hazards (Chan and Rhodes 2013)
Individual well-being—with a focus on adaptive behaviour, health, and compassion	Ability to cope with stressful situations Cognitive flexibility and adaptive behaviour Psychological and physical health Empathy, compassion for others ↔ reduction of compassion fade Human–nature connection/nature connectedness Capacity for minimizing automatic, habitual, or impulsive reactions Activation of core values/empowerment	Links with climate adaptation are mainly indirect Little attention paid to health effects of climate change and, consequently, prevention, preparedness, etc. of these effects through, for instance, mindfulness (e.g., Costello et al. 2011)
Organisational management—with a focus on organisational reliability and innovation	Organisational mindfulness Mindful organising/organising (for) mindfulness Workplace mindfulness Compassion organising/organisational compassion/compassionate decision-making Organisation’s corporate philanthropic disaster response Resilience performance Corporate social responsibility/social entrepreneurship Sacred activism Organisational learning Job satisfaction and performance Good leadership and decision-taking Moral identification/moralised and ethical leadership (cf. Fehr et al. 2014)	Not linked to adaptation-related frameworks, such as adaptation policy integration/mainstreaming (Wamsler 2014) Organisational concepts require further construct validation and empirical testing regarding its responsiveness to interventions (Carroll and Rudolph 2006; Vogus 2011; Thomas et al. 2015) Potential negative impacts of compassion have been little assessed (Simpson et al. 2014a, b) The mindfulness term is, in parts, used quite loosely, without close linkages to broader issues (e.g. origins, inner change processes) While there are studies that show the importance of special leadership capacities for managing unpredictable and/or extreme events (Paté-Cornell and Cox 2014; Kapucu and Van Wart 2008) and the importance of mindfulness for good leadership, there are hardly any studies that look into the interface of both aspects
Environmental behaviour—with a focus on ecological well-being and resilience	Nature connectedness, compassion for the environment Pro-environmental values Pro-environmental intentions and engagement Mindful consumption and sustainable lifestyle Sustainability-oriented innovations Environmental justice Erosion of mindfulness/culture (linked to vulnerability) Personal-to-planetary well-being, ecopsychology, animism	Focus is on climate mitigation, not climate adaptation (Wamsler et al. 2017), e.g., research on well-being and ecologically responsible behaviour In the context of climate adaptation, there is a lack of critical consideration of how mindfulness could counteract maladaptation (e.g., in urban planning) The term mindfulness is, in parts, used without close linkages to its origins (e.g., mindful consumption). More critical approaches are often missing
Social justice—with a focus on social activism and change	Political mindfulness Social and sacred activism Mindful social change Mindful engagement—do no harm principle Moral awareness/judgement Compassion for others Non-judgemental attitude Activation of core values/empowerment	Few scholarly articles/studies that explicitly address the mindfulness–adaptation nexus More practice-based advancements with, so far, little empirical evidence (e.g., mindful social change)
Knowledge production—with a focus on more holistic research	Deep listening Mindful engagement Mind–body distinction/connection Ontological hybridity—cross-hybrid learning Non-material paradigm/causation	Few scholarly articles/studies that explicitly address the mindfulness–adaptation nexus and its implications for research approaches and methodologies There is a disconnect between science and human experience that does not capture the holistic picture necessary for adaptation to, and resilience in the face of, climate change (cf. Knodel 2012; Gibbson and Wisner 2016)
Across the six research fields	Compassion-related studies are creating implicit links between mindfulness and climate adaptation	Few linkages between the different research areas and across-scales Little attention is given to proactive adaptation and risk reduction Almost no targeted research on climate adaptation and mindfulness

**Table 2:** Examples of identified research focus areas in relation to different adaptation phases/contexts

Adaptation phase/context	Researched links (selected examples)	Related gaps
Response and response preparedness	Mindfulness-based interventions to increase psychological resilience: e.g., psychological first aid, and integration of care in disaster relief work, self-care after disasters	Lack of research on response preparedness Organisations are generally unequipped to provide assistance to increase psychological resilience, such as mindfulness-based approaches, for disaster victims and emergency workers (e.g., Ehrenreich and Ellicot 2004; Carroll et al. 2009) Lack of coherent, systematic monitoring of the impact of climate/natural hazards on people’s health and well-being and associated support to mitigate and manage the effects (Carroll et al. 2009) There is a need for a more complex analysis of, and response to, the psychosocial processes following a disaster (Cox and Perry 2011)
Recovery and recovery preparedness	Recovery of sense of self, meaning, and community Compassion-based organisational responses to assist disaster-affected employees Post-traumatic stress reduction during recovery Role of communities of faith and faith-based organisations during disaster recovery Well-being, health, cultural and religious values and their impact on communities’ recovery Importance of mindfulness for post-traumatic growth	Lack of research into recovery preparedness (e.g., to prepare for the return to work following hazards/disasters) Potential role of nature-based adaptation for recovery and vulnerability reduction has, so far, hardly been discussed (e.g., regarding psychological and physical health) Longer term studies (e.g., on the effects of mindfulness on post-traumatic growth) are missing (Shiyko et al. 2017) The urgent drive for recovery and rebuilding can obscure important social-psychological processes needs that can undermine long-term sustainability and community resilience (Cox and Perry 2011)
Development (adaptive capacity and proactive risk reduction)	Adaptive behaviour: ability to generate varied responses to the same stimuli and decreased automatic and habitual responses Reduced cognitive rigidity and increased creative thinking crucial for climate change context and associated uncertainty Social justice Role of change agents	The health effects of climate change receive a little attention from climate scientists (Costello et al. 2011) While there is a consensus that risk is socially constructed, there are hardly any studies that look into the issue of mindfulness and social justice in climate adaptation
Vulnerability and climate change context	Well-being, health, cultural, and religious values and how they impact the ways communities interpret risk and climate change, and how they approach risk reduction Impact of disasters on people’s well-being, which becomes an issue of vulnerability, e.g., in relation to health, family cohesion Social justice, pro-social responses Degradation of mindfulness as an aspect of people’s vulnerability facing climate change, linked to indigenous knowledge Visible environmental degradation (e.g., through climate hazard) impacts health and is associated with increased physical illness and declining mental health, e.g., provoking depressive symptoms and a loss of sense of place, which, in turn, can be addressed by mindfulness	The health effects of climate change have received relatively little attention from climate researchers (Costello et al. 2011) The abstract nature of climate change makes it non-intuitive and cognitively effortful to grasp (Markowitz and Shariff 2012) While there is a vast amount of literature that confirms the importance of emotion, affect and power for risk perception, risk assessment, risk and communication, and action-taking (e.g., Slovic 1999), potential approaches to address these aspects (such as mindfulness) have been little studied

**Table 3:** Examples of research focus areas and their relation to different adaptation scales

Scale	Researched links (selected example)	Related gaps
Individual level	Mindfulness may increase human potential, e.g., through stress reduction and compassion	Cognitive and agent-based focus of research with few links to structural, systemic change Few empirical studies and explicit links to climate change and adaptation
Organisational level	Mindfulness may increase collective human potential, e.g., through improved leadership, organisational learning, and compassion for others within and outside the organisation	Studies focus on cognitive processes in high reliability organizations and associated managerial aspects within organisations, but a little enquiry into their societal impacts and broader structural change (e.g., through upscaling/mainstreaming) Few explicit links to climate change and adaptation
Societal level	Mindfulness may increase action-taking for the common good, both individually and collectively	In contrast to the individual and the organisational level, there is hardly any research at the societal level Related networks, scholarship etc. are just emerging/in the making
Science/research across levels	Mindfulness-based approaches may support more holistic research on disaster and climate risk reduction	In contrast to the individual and the organisational level, there is hardly any research that deals with epistemological and ontological questions

### Disaster management and well-being research

The few studies that explicitly link mindfulness with climate adaptation mostly lie at the interface between well-being and disaster management research (Tables 1, 2) with a focus on the post-disaster context (Table 2), and at an individual scale (Table 3). It is a growing body of research that illustrates the importance of mindfulness in post-disaster response and recovery to increase psychological resilience. Studies assess the potential importance of faith-based coping (Chen n.d.; Cherry and Allred 2012; Henslee et al. 2015; McGeehan 2012) and mindfulness interventions for different target groups: disaster victims, disaster aid workers (e.g., firefighters, healthcare professionals^1^ and volunteers), and disaster researchers (Catani et al. 2009; Hechanova et al. 2015; Hoeberichts 2012; Matanle 2011; Srivatsa et al. 2013; Yoshimura et al. 2015; Zeller et al. 2015; Eriksen and Ditrich 2015; Smith et al. 2011; Waelde et al. 2008; Setti and Argentero 2015, 2014; Abeni et al. 2014; Dierynck et al. 2017; Raab et al. 2015). They show the positive influence not only for reducing post-disaster trauma, post-traumatic stress disorder,^2^ and compassion fatigue, but also for supporting post-disaster growth (e.g., Chan and Rhodes 2013; Jacobsen 2008; Hanley et al. 2015; Shiyko et al. 2017).^3^ These beneficial effects of mindfulness and associated attributes are said to be related to its influence on well-being, in particular: (1) the ability to cope with stressful situations; (2) psychological/cognitive flexibility to adapt to new circumstances (Brown et al. 2007; Hechanova et al. 2015; Moore and Halinowski 2009; Waelde et al. 2008); (3) the capacity to minimize automatic, habitual, or impulsive reactions (Brown et al. 2007; Hechanova et al. 2015; Hülsheger et al. 2013; Waelde et al. 2008); (4) psychological and physical health in general (Black and Slavich 2016); (5) the activation of core values and compassion for others and the environment (Ericson et al. 2014; Howell et al. 2011, 2013; Lockhart 2011); and (6) the creation of a sense of self and place (Dueck and Byron 2011; Hanley et al. 2015; Roberto et al. 2010; cf.; Vandemark 2007) (Table 1).

While these capacities are clearly crucial in all phases and contexts of climate adaptation (Table 3), there are almost no studies of the interface between well-being and disaster management that address the issues of: (1) climate change; (2) mindfulness disposition; and (3) proactive adaptation, including response and recovery preparedness (Ryan 2016; Wamsler et al. 2017).^4^ The above-mentioned survey by Wamsler and Brink (2018) fills this gap by exploring how individual mindfulness is linked to climate adaptation. It complements the existing literature by showing that mindfulness disposition (as opposed to mindfulness interventions) might have positive impacts on response, recovery, and proactive climate adaptation. More specifically, a high mindfulness score was found to be positively correlated with a high score for overall motivation for climate adaptation, including both pro- and reactive actions (Wamsler and Brink 2018). In addition, while the number of adaptation actions taken was not correlated to individual mindfulness, a positive correlation was found between mindfulness disposition and one adaptation action, namely warning neighbours about a storm (Wamsler and Brink 2018). This aspect relates to another gap identified in the literature.

In fact, disaster management research that addresses the mindfulness–adaptation nexus has vastly overlooked issues of risk perception and risk communication, and how they translate into action-taking (Table 1). Only studies can be found that focus on certain attributes of mindfulness, such as emotions and compassion (e.g., Roeser 2012; Lu and Schuldt 2016; Smith and Leiserowitz 2014). Based on a diverse American sample, Lu and Schuldt (2016) show for instance that higher levels of compassion are consistent with a stronger belief that climate change is caused by human activities which, in turn, can mediate increased policy support. Positive emotions and compassion-based climate change communication have also been found to be more effective than ‘motivation by fear’ in the context of extreme weather and other climate phenomena (O’Neill and Nicholson-Cole 2009; Ryan 2016; Smith and Leiserowith 2014). In addition, Moser (2008) found that a sense of meaning beyond self-serving goals is essential in countering an individual’s sense of isolation and futility vis-a-vis global warming. Nevertheless, many knowledge gaps remain (Coombs 2009; Roeser 2012; Lu and Schuldt 2016; Bohensky and Leitch 2014).

### Disaster management and organisational research

The interface between disaster management and organisational research is another area that has addressed increasingly the mindfulness–adaptation nexus in recent years (Table 1; Levinthal and Rerup 2006). This is reflected in the emergence of concepts^5^ such as organisational mindfulness (Becke et al. 2012), mindful organising (Vogus 2011), organising (for) mindfulness (Thomas et al. 2015; Vogus 2011; Vogus and Sutcliffe 2012; Weick and Putnam 2006), workplace mindfulness (Dane and Brummel 2014), compassion organising (Duton et al. 2006; Shepherd and Williams 2014; Simpson et al. 2015), organisational compassion (Simpson et al. 2015); compasionate decision-making (Simpson et al. 2014a, b); resilience performance (Weick and Sutcliffe 2007), social entrepreneurship (Fernando 2007; Miller et al. 2012), corporate philanthropic disaster responses (May et al. 2015; Miller et al. 2012), and sacred activism (Pio and Syed 2014). These concepts describe individual and collective social and cognitive processes that increase a capability for awareness and support organisational learning and adaptability in a context of insecurity and uncertainty (Chiva and Habib 2015; Levinthal and Rerup 2006).

Organisational mindfulness emerged from the domain of risk and safety research, and has only recently been extended to climate adaptation (Aviles and Dent 2015; Becke 2014; Becke et al. 2012; Senghaas-Knobloch 2014). It describes behaviours of collective mindfulness in high reliability organisations (e.g., disaster management) that help in adapting to unexpected events (Weick et al. 1999). Organisational mindfulness is said to support collective and organisational learning with respect to the anticipation of, and coping with, unexpected events (Becke et al. 2012; Becke 2014), and to actively nurture the key social resources that underlie performance and sustainability when facing climate events with potentially long-lasting consequences (Becke 2014). It is based on the stated positive influence of mindfulness on overall well-being, understanding complexity, sensitivity to context and multiple perspectives, as well as cognitive flexibility and openness to novelty that, in turn, encourage organisations to constantly probe their environment for ways to stay ahead through innovation (Brown and Eisenhardt 1997; Greenberg et al. 2012; Colzato et al. 2012; Vogus and Welbourne 2003; Weick et al. 1999). In contrast, the notions of ‘compassion organising’ and ‘corporate philanthropic disaster response’ focus on how organisations relate to employees who have been affected by natural hazards (Simpson et al. 2015; Watkins et al. 2015; Thomas et al. 2015). Given the mounting evidence regarding differential vulnerability and the different impacts of hazards and associated responses, such approaches are becoming increasingly relevant for both organisational reliability and social equity (Hochwarter et al. 2008; Lilly et al. 2008).

Most of the organisational management concepts that address the mindfulness–adaptation nexus have only emerged in recent years, and empirical studies that provide evidence and practical guidance remain rare (Carroll and Rudoph 2006; Thomas et al. 2015; Table 1),^6^ especially in relation to climate adaptation. In addition, there is a lack of links between these concepts and organisational frameworks relevant to climate adaptation, such as adaptation policy integration and mainstreaming (Wamsler 2014). Another gap in the research is the lack of assessments of the potential negative impacts of mindfulness and compassion-based approaches in organisational management. As such approaches relate to complex social processes, embedded within power relations, outcomes are more likely to be ambivalent than wholly positive (Simpson et al. 2014a, b; Table 3).

This links to another area of organisational research relevant for exploring the mindfulness–adaptation nexus, namely leadership. In addition to the qualities that are traditionally associated with good leadership (vision, authority, etc.), mindfulness and associated emotional intelligence are increasingly seen as crucial (Goleman 1998, 2011). Studies have looked at the role of mindfulness (and mindlessness^7^) in good (bad) leadership and decision-making, as a component in leadership processes, as a leader attribute, and/or a human resource practice (Goleman 1998, 2011; Marques 2014; Vogus 2011; Vogus and Welbourne 2003). This is especially crucial in a context of climate change, where poor leadership has been shown to maximize hazard impacts, seen in the example of the Katrina–Rita hurricanes in New Orleans (Paté-Cornell and Cox 2014; Kapuco and Van Wart 2008), and where mindlessness has been associated with maladaptation, both in psychological and physical terms (Langer 1989). The survey results of Wamsler and Brink (2018) support the outcomes of this literature review in that they show that a high mindfulness score was found to be positively correlated with a high score for overall motivation to support or engage in adaptation. No further outcomes could be derived regarding organisational management, since the survey focused on at-risk-citizens, and not on climate professionals and/or organisations (Wamsler and Brink 2018).

### Environmental and well-being research

In the domain of environmental research, fewer studies could be identified that make an explicit mindfulness–adaptation link (Table 1). Most environmental studies focus on objective interactions between natural, social, and human systems, while subjective, human, aspects tend to be ignored (Sumi 2007). However, it is a rapidly growing field. In fact, there is an increasing number of studies at the interface between environmental and well-being research that provide evidence for the potential influence of mindfulness on issues such as (1) human-nature connectedness (Amel et al. 2009; Anthony 2013; Howell et al. 2011; Lockhart 2011), (2) compassion for the environment (Pfattheicher et al. 2016; Bai 2013), (3) pro-environmental values (Ericson et al. 2014), (4) intentions and engagement (Amel et al. 2009; Brown and Kasser 2005; Jacob et al. 2009; Pfattheicher et al. 2016; Siqueira and Pitassi 2016), and (5) sustainability-oriented lifestyles and innovations (Amel et al. 2009; Brown and Kasser 2005; Brown and Ryan 2003; Brown et al. 2007, 2004; Ericson et al. 2014; Goleman 2009; Jacob et al. 2009; Sheth et al. 2010; Siqueira and Pitassi 2016; Lengyel 2015). Importantly, these aspects can assist in overcoming key psychological barriers that are said to limit climate adaptation, such as risk perceptions, anxiety, compassion fade, and ideological worldviews that preclude pro-environmental attitudes and behaviours (Gifford 2011; Markowitz et al. 2013; Moser 2008). Nevertheless, psychological aspects of climate change and adaptation have so far barely hit the radar of climate change science.

This situation has also given rise to the burgeoning ecopsychology movement. Ecopsychology can be seen as a psychological viewpoint of animism (Bai 2013; Reser and Bragg 2013). It seeks to understand ways to expand the emotional connection between individuals and the natural world and, ultimately, support more sustainable behaviour. Accordingly, it is interested in the knowledge provided by a wide variety of ancient and modern cultures that have a history of embracing nature, and its scholars argue that mindful awareness of our interdependence with nature not only helps us to regain our lost, ecologically embedded identity, but also helps us to behave more sustainably (Amel et al. 2009; Mayer and Frantz 2004; Roszak 1992; Roszak et al. 1995). This understanding is supported by an expanding body of scholarly evidence, suggesting that mindfulness is associated with environmentally responsible behaviour. It also led to the notion of ‘ecological mindfulness’, which was put forward in 2015 (Mueller and Greenwood 2015; Sol and Wals 2015). The survey by Wamsler and Brink (2018) supports this literature review in that a positive correlation was found between mindfulness and being a vegetarian.

The understanding that personal and planetary well-being are intrinsically interlinked (Koger 2015; Roszak et al. 1995; Jacob et al. 2009; Brown and Kasser 2005) is also in line with deep ecology’s contention. It says that humankind requires a fundamental shift in consciousness to ensure ecological sustainability, and that the subject/object dualism inherent in Western empiricism and materialism will need to be transcended by meditative approaches such as mindfulness (Brinkerhoff and Jacob 1999). This resonates with the mindfulness principle of dependent origination (interdependence and interpenetration), which recognises that all beings are deeply connected to other beings and the world, including their actions and thinking (Yeh 2006; Bai 2013).

Most studies at the interface between environmental and well-being research address the mindfulness–adaptation nexus implicitly, for instance by focusing on compassion, an attribute of mindfulness (Table 1). They show that compassion can be positively correlated with pro-environmental values and behaviours that can translate into increased policy support, for example, in the form of donations to environmental organisations (Dickinson et al. 2016; Pfattheicher et al. 2016). At the same time, compassion fade has been found to be negatively correlated with pro-environmental values and behaviours, which may challenge the collective ability and willingness to confront major environmental problems (Markowitz et al. 2013). Compassion fade is said to reduce environmental identity, and is a significant psychological barrier to building broad public support for addressing environmental problems such as climate change (Markowitz et al. 2013).

### Environmental and social justice research

Social justice is another relevant field for exploring the mindfulness–adaptation nexus (Table 1). Climate change has environmental and health consequences that disproportionately affect low-income countries and poor people in high-income countries, who must be protected (Levy and Platz 2015; Costello et al. 2011). It threatens civil and political rights, including the right to life, access to safe food and water, health, security, shelter, and culture (Levy and Platz 2015). Naturally, climate adaptation has become an issue of equity and social justice.

Mindfulness is said to politically sensitize people and organisations to the consequences of unquestioned structures and power relations at all scales (Dayley 2017; Senghaas-Knobloch 2014; Rowe 2017; Wamsler et al. 2017). It implies awareness and sensitivity to context, the cultivation of compassion, and intrinsic core values which, in turn, are assumed to be reflected in actions for the common good (Brown and Kasser 2005; Ericsson et al. 2014; Hanh and Weisman 2008; Kaza 2008). Accordingly, mindfulness does not simply address cognitions and cognitive schemes, but also fosters a sense of appropriate, or just, behaviour (Dayley 2017; Dhiman and Marques 2016; Senghaas-Knobloch 2012; Wamsler et al. 2017). However, associated empirical research has focused mainly on issues such as pedagogy (e.g., the integration of mindfulness into anti-oppressive pedagogy) and social work (e.g., the integration of mindfulness into social justice approaches into social work) (Berlila 2016; Hick and Furlotte 2009), not on climate adaptation.

Research into the interface between mindfulness and social justice in a context of climate change remains rare. However, it is becoming the focus of current mindfulness debates (Davis and Kabat-Zinn 2015) and a range of new concepts, such as political mindfulness (Rowe 2017; Senghaas-Knowbloch 2012, 2014), sacred activism (Pio and Syed 2014), mindful engagement (Wood and Mazur 2016), mindful social change^8^, and associated studies of moral awareness and judgement (Markowitz and Shariff 2012; Senghaas-Knowbloch 2012) are emerging. ‘Political mindfulness’ is closely related to organisational mindfulness (Table 1), but with a focus on the broader political community that sets the norms and rules for societal development, e.g., inter- and transnational organisations such as the World Bank (Senghaas-Knowbloch 2012). In contrast, sacred activism takes a bottom–up approach. A sacred activist can be described as someone who intentionally works towards social change, by providing compassionate services for others in the face of growing injustice, suffering, and violence, based on the perception that the world is experiencing a profound crisis and everyone needs to act (Harvey 2009; Pio and Syed 2014). Sacred activism is also closely linked to organisational research, as it is understood to be intertwined into a practical and pragmatic drive to transform institutions (Pio and Syed 2014). Similarly, ‘mindful engagement’ and ‘mindful social change’ concepts are emerging. Mindful engagement looks at collective social and environmental responsibility, based on the humanitarian principle of ‘do no harm’ (Anderson 1999; IFRC 2011; Wood and Mazur 2016). Mindful social change aims to explore the connection between mindfulness and transformative social change (Gonzáles 2017). However, here again, scholarly research is scarce and almost non-existent in relation to climate adaptation.

The complementary survey by Wamsler and Brink (2018) adds to this literature review in that it shows linkages between mindfulness and ‘other-focused’ social factors. More specifically, it showed a positive correlation between individual mindfulness and being motivated by social factors (i.e., encouraged by friends and family, reducing the risk for others, and having a ‘good conscience’), while economic and ecological motivation could not be linked to mindfulness (Wamsler and Brink 2018). This outcome can be linked with social equity and justice. In addition, while the number of adaptation actions taken was not correlated to mindfulness disposition, a positive correlation was found between mindfulness disposition and one ‘other-focused’ adaptation action, namely warning neighbours about a storm (Wamsler and Brink 2018).

### Knowledge production

Finally, there is one other emerging body of research that addresses the mindfulness–adaptation nexus, namely knowledge production. This can be seen as a reaction to the increasing recognition of a disconnect between human experience and scientific activity that does not capture a holistic picture of climate adaptation, risk reduction, and resilience for instance (Bai 2013; Gibson and Wisner 2016; Knodel 2012, 2014). Science has always been shaped by contemporary problems, and it evolves with them. Until now, science research has been dominated by reductionism, as the intellectual and social model (Bai 2013). However, successful, it has been in the past, emerging policy issues and research into neuroplasticity, emotions and mindfulness seem to suggest that this ideal of rationality is no longer appropriate (cf. Schwartz 2011; Wamsler et al. 2017). Accordingly, risk and climate scholars are increasingly calling for research that incorporates local knowledge, i.e., ways of being, thinking, and knowing that are informed by centuries of lived experience and culture, and an approach that involves deep listening and joint internal reflection through external engagement (Gibson and Wisner 2016; Knodel 2012, 2014). This understanding is consistent with ecopsychology, animism, ecological mindfulness (Table 1), and associated interdisciplinary and cross-hybrid learning (Chinn 2015; Mueller and Greenwood 2015; Sol and Wals 2015). The latter states that knowledge production requires spaces where different perspectives can take root, be nurtured, and flourish into ways of knowing, being, and becoming that serve people, places, and the planet (Greenwood 2013; Gugerli-Dolder and Frischknecht-Tobler 2011; Sameshima and Greenwood 2015). It requires more expansive and inclusive approaches where researchers are free to take risks—risks that cannot be taken when academic fiefdoms determine the questions that are asked and regulate methodologies, rather than encourage new thinking and creativity (Mueller and Greenwood 2015). Such an understanding also resonates with recent feminist and cultural geography literature that speaks of emotion as an element in choosing research questions, and a driver of how research is conducted (Davidson et al. 2012; Sharp 2009).

However, only few studies have actually attempted to develop better approaches to knowledge production regarding risk and adaptation. An exception is the Frontline method, developed by the Global Network of Civil Society Organisations for Disaster Reduction (GNDR) (Gibson and Wisner 2016). This is based on mindfulness approaches such as deep listening (Hanh and Weisman 2008). This kind of ‘listening’ goes beyond the linguistic (i.e., language) and the cognitive (i.e., the application of frameworks derived from theories), and refers to opening up emotionally to an interlocutor, acknowledging one’s emotional reaction to the situation one is studying, and allowing a transformational process of internal reflection through external engagement (Knodel 2012, 2014; Gibson and Wisner 2016; Wisner 2015).

Overall, the mindfulness–adaptation nexus raises questions about epistemological and ontological frameworks, the materialist paradigm that has shaped the construction of knowledge, in general, and climate science, in particular (cf. Bai 2013; Osborne and Grant-Smith 2015; Schwartz 2011; Schwartz et al. 2005; Wamsler et al. 2017). Some scholars argue that the current mind–body distinction itself may represent a premature cognitive commitment (Langer 1989), which requires that non-material causation need to be recognised as part of scientific inquiries (Bai 2013; Wamsler et al. 2017; Schwartz 2011; Schwartz et al. 2005). Here, this also means exploring the issue of mindfulness in climate adaptation, despite resistance and scepticism from scholars who dismiss the importance of critically looking at sustainability from an angle that is very different to the classical, large-scale, political, or institutional perspective.

## Discussion and conclusions

The increasing numbers of disasters and climate change mean that humanity is facing ever more complex challenges (Kates et al. 2001; Sol and Wals 2015). However, the current approaches appear insufficient to address them (Kunze 2012; Sol and Wals 2015). New narratives and social practices are called for that encourage a broader, cultural shift towards sustainability (Dhiman and Marques 2016; Esbjörn-Hargens 2009; Gidley 2007; Inglis 2008; Frawley 2006; Wamsler et al. 2017). This study shows that mindfulness has the potential to support such a shift. While current knowledge on mindfulness in climate adaptation remains scarce and fragmented (cf. Tables 1, 2, 3), it is gaining increasing momentum. Nearly all the relevant literature has been published in the past 5–10 years.

Mindfulness and adaptation are more connected than is generally thought. Based on an in-depth literature review, this study shows that mindfulness has the potential to contribute to facilitating climate adaptation at all scales, from the individual to the institutional and societal level. It may increase individual and collective capacity to deal with increasing risk and uncertainty—through cognitive, emotional, managerial, structural, ontological, and epistemological change processes. However, many research gaps remain (cf. Tables 1, 2, 3).

The analysis of the mindfulness–adaptation nexus resulted in the identification of several core conceptual trajectories for mindful climate adaptation (Tables 1, 2, 3); they imply the critical consideration of mindfulness in supporting:

1. Private^9^ adaptation: for instance, by reducing vulnerability (e.g., psychological and physical well-being, and risk perception), improving post-disaster response, recovery, and growth (e.g., the ability to cope with stressful situations), and increasing motivation and action-taking for reducing risk (e.g., clarification of values, increased empathy and compassion, adaptive capacity, and environmental behaviours).
2. Public–private adaptation and governance: for instance, by improving climate change communication, climate policy support, and new social approaches, norms, and values that challenge the business-and-power-as-usual norm. Mindfulness can thus be seen as another pillar in institutional attempts to support transformation, which can complement other angles. Criticism of existing institutions and power relations as drivers of vulnerability and risk thus also need to be extended to include a critique of these institutions as inflexible, unimaginative, and emotionally dead [classically seen as the characteristics of bureaucracy (Fesler 1965)].
3. Adaptation policy integration and mainstreaming: for instance, by influencing organisational reliability (organisational learning and innovation), nurturing social capital (good leadership and staff support), providing an ethical grounding, and a legitimate basis to negotiate adaptation objectives across cultures and inspire better practices (compassion for others, social activism, equity, and justice).
4. Adaptation science: for instance, by shaping new research questions, methodologies (deep listening, cross-hybrid learning, non-material causations) and, ultimately, knowledge production. This requires the incorporation of local knowledge, acknowledging and respecting humanity (including citizens, bureaucrats and even corrupt leaders), possibly leading to dialogue and positive change.^10^

Finally, in terms of the four dimensions that are identified, mindful climate adaptation also implies: (1) the critical consideration of their interdependence (e.g., individual and planetary well-being and adaptation), and (2) a detailed evaluation of potential drawbacks (Doran 2017; Walsh 2016). While this study did not find any structural critiques of mindfulness for climate adaptation, potential side-effects (Howard 2016), the inappropriate use of techniques (Williams and Kabat-Zinn 2011; Purser and Loy 2013), and the potential co-optation of mindfulness for capitalist purposes (Carrette and King 2005) have to be considered. In addition, further research is needed to critically look into context and pre- or co-conditions that can support individual mindfulness to bring about societal transformation. This sudy concludes that mindfulness has the potential to foster climate adaptation at multiple levels, from individual to global, and calls for further critical research into how we can tap into this potential to drive global change.

## Footnotes

1 Several reviews can be found on the potential of mindfulness-based approaches for health care professionals such as Irvin et al. (2009) and Raab (2014).

2 Post-traumatic stress disorder (PTSD) is an anxiety problem that may develop in some people after exposure to extremely traumatic events, such as climate hazards/disasters. Kim et al. (2013) review 16 publications and find that mind–body practices (including mindfulness-based practices) are associated with positive impacts on PTSD symptoms.

3 See Collins and Long (2003) and Thompson et al. (2011) for related reviews.

4 An exception is Lyles (2015), who looks into the potential of applying ‘compassion building programs’ for sustainable planning in general, and risk reduction and adaptation planning in particular.

5 Such concepts relate either directly to mindfulness or indirectly, in terms of compassion. Scholars define compassion in organisations as a threefold relational process of: collectively noticing that another is suffering, empathising with their pain, and responding.

6 An exception is the development of games (and associated research) designed to improve compassionate responses developed by scholars and staff at the USAID Center for International Disaster Information (Özpolat et al. 2015).

7 Brown and Ryan (2003) consider mindlessness as the relative absence of mindfulness, which, in simple terms, is found when individuals behave compulsively or automatically, without awareness of or attention to their behaviour (Deci and Ryan 1980).

8 This new field is manifested in the creation of professional networks (e.g. the Mindfulness Initiative and its Mindfulness and Social Change Network, established in 2014), and academic journals (e.g. the *Journal of applied thought leadership in operation, process improvement, people development, and mindful social change*).

9 ‘Private adaptation’ refers to initiatives taken by individuals or private households, rather than authorities or governments, and corresponds to ‘autonomous adaptation’. Both private/individual and public adaptation can be a conscious or unconscious response to actual or expected climate change, including variability and extremes (IPCC 2001, 2014; Malik et al. 2010).

10 This implication is especially relevant for those who support criticisms of the political ecology (cf. Wisner 2015).
